# Glycomics for Microbes and Microbiologists

**DOI:** 10.1128/mBio.01224-16

**Published:** 2016-08-09

**Authors:** Peter N. Lipke

**Affiliations:** Biology Department, Brooklyn College of the City University of New York, Brooklyn, New York, USA

## Abstract

The recent article “Lectin-Glycan Interaction Network-Based Identification of Host Receptors of Microbial Pathogenic Adhesins” by Ielasi et al. describes a new development in microbial carbohydrate analysis [Ielasi FS, Alioscha-Perez M, Donohue D, Claes S, Sahli H, Schols D, Willaert RG, mBio 7(4):e00584-16, 2016, http://dx.doi.org/10.1128/mbio.00584-16]. Specific carbohydrate ligands have been identified from the patterns of lectin binding to oligosaccharides printed on a chip. The new technique links the output to a comprehensive glycan database and offers a number of data visualization options. The graphs highlight the occurrence of potential ligands, organized by organism, tissue, and patterns of association with disease states. The analysis has successfully predicted novel glycoprotein ligands for microbial lectins, including an interaction of *E. coli* FimH with HIV gp120.

## COMMENTARY

Lectins are nonenzyme proteins that bind to specific glycan determinants. Lectins displayed on cell surfaces act as cell adhesion proteins. Many microbial surface lectins initiate host-microbe or microbe-microbe interactions leading to commensal or disease states. Indeed, lectin binding can initiate signaling pathways that effect cellular functions, including innate and acquired immune responses (1–5).

**FIG 1:**
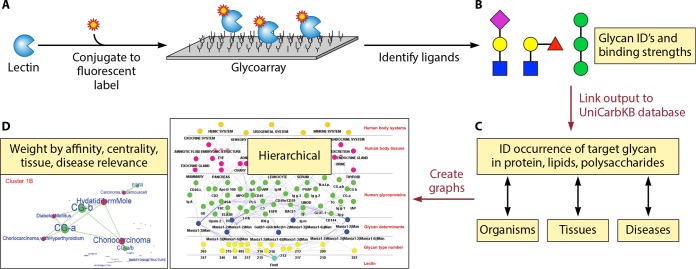
Workflow described in Ielasi et al. (10). (A) A fluorescently labeled microbial lectin is used to scan a glycochip printed with ~500 individual oligosaccharides, and the extent of binding is monitored. (B) Target oligosaccharides are depicted as colored shapes according to a graphic convention, where a circle is a hexose, a square is an aminosugar, etc., and each color represents a specific configuration, e.g., galactose is yellow and mannose is green (http://www.functionalglycomics.org/static/consortium/Nomenclature.shtml). Ielasi et al. (10) have used this output to datamine UniCarbKB (C), a curated database for glycan structure, occurrence, and association with specific tissues, diseases, and conditions. (D) A set of graphing routines highlights these associations, predicts ligands, and potentially will identify oligosaccharides that would be competitive inhibitors of the lectin. The bioinformatic processes developed in the paper are depicted by dark red arrows and labeled in the same color. ID, identification/identify.

It is a long and arduous experimental path from the identification of a lectin to the determination of its roles in cell signaling, cellular differentiation, and host interactions. The first step is often the identification of ligands, and this step has become much more systematic with glycochip technology (Fig. 1A and B). The lectin protein is isolated, tagged with a fluorescent reporter, and then incubated on a slide imprinted with ~500 oligosaccharides, each with a known structure and specific position in the array. The pattern of fluorescent spots due to lectin binding is used to deduce the binding specificity of the lectin. Each lectin belongs to broad structural and functional classes named for the major monosaccharide recognized (e.g., mannose specific or galactose specific), but each lectin has a slightly different binding specificity, depending on the size and structure of its individual binding site.

Several groups have reported glycochip analyses of binding specificity for several microbial lectins, including FimH from *Escherichia coli*, Als1 and Als3 from *Candida albicans*, and Epa galectins from *Candida glabrata* (6–9). This much is standard. However, the Willaert group now has taken the results two steps farther (10). First, they have developed software that uses the glycochip results to query UniCarbKB, a comprehensive database of lectins and their ligand glycans (Fig. 1C). The database is sortable and searchable by structure, taxonomy, tissue, protein, or associated disease. The authors have used these data to create weighted graphs with nodes displaying the occurrence of a ligand in specific glycoproteins of organisms and their tissues, connections to other potential ligands and lectins, and association with disease states (Fig. 1D). They have chosen to weight the nodes by their connectivity (the number of related systems, which is an index partially determined by the frequency of occurrence of the node in the literature covered in the database). The lines connecting the nodes (edges) are weighted by binding strength and by the frequency with which the nodes have been associated with each other in the literature (Fig. 1C and D). The software is available through an FTP site listed in the supplemental materials of the paper (10).

The consequences of such analyses illustrate the power of the bioinformatics approach. Some of the predicted interactions include *C. albicans* Als3 binding to glycoproteins containing β-linked *N*-acetyl glucosamine residues and interactions of the *C. glabrata* Epa lectins with β-galactose-containing glycans. Ielasi et al. confirmed these predictions by demonstrating binding to specific glycoprotein targets and by showing inhibition of binding by monosaccharide competitors (10). The links through UniCarbKB highlight the occurrence of ligands in glycoproteins and glycolipids, especially those whose expression is increased in specific tumors or other diseases (Fig. 1D). These results are potentially useful to predict increased probability of infections in victims of the specific tumor types identified. In addition, the lectins may be assessed as potential vaccine immunogens, and their ligands can be assayed as inhibitors of microbial binding (11).

The most spectacular result reported by Ielasi et al. is the identification of HIV gp120 as a ligand for FimH, with a dissociation constant in the equilibrium state (*K_D_*) of <1 µM. FimH is well documented as a lectin specific for mannose-containing glycans with α1,2 and α1,3 linkages (6), and previous work has focused on ligands in human tissues. The UnicarbKB query from the FimH glycochip output identified viral glycoproteins, including HIV gp120, as potential ligands, interactions not documented in PubMed. The authors used surface plasmon resonance (SPR) to confirm recombinant FimH binding to gp120 with *K_D_* values below 1 µM. Furthermore, the FimH lectin domain inhibited HIV-1 replication in assays using MT4 lymphocytic cells, peripheral blood mononuclear cells, and HIV indicator line TZM-bl. The 50% effective concentrations were 20 to 70 µg/ml for two strains of HIV. This result, spectacular in both its novelty and potential application, is the type of interaction uncovered using this methodology.

The featured paper describes a breakthrough in functional glycomics. The technique links specific glycan ligands to laboratory-based and disease-based knowledge through UniCarbKB. The resulting graphs have shown (and the authors have validated) potential microbial interactions with mammalian hosts and with viral glycoproteins. Thus, there is now a systematic way to link experimental results to the vast volume of acquired knowledge in glycobiology.
